# Time Trends in Blood Pressure, Body Mass Index and Smoking in the Vietnamese Population: A Meta-Analysis from Multiple Cross-Sectional Surveys

**DOI:** 10.1371/journal.pone.0042825

**Published:** 2012-08-09

**Authors:** Quang Ngoc Nguyen, Son Thai Pham, Viet Lan Nguyen, Lars Weinehall, Ruth Bonita, Peter Byass, Stig Wall

**Affiliations:** 1 Department of Cardiology, Hanoi Medical University, Hanoi, Vietnam; 2 Vietnam National Heart Institute, Bach Mai Hospital, Hanoi, Vietnam; 3 Department of Public Health and Clinical Medicine, Umeå Centre for Global Health Research, Umeå University, Umeå, Sweden; 4 School of Population Health, Faculty of Medical and Health Sciences, University of Auckland, Auckland, New Zealand; Tulane School of Public Health and Tropical Medicine, United States of America

## Abstract

**Introduction:**

Data for trends in cardiovascular disease (CVD) risk factors are needed to set priorities and evaluate intervention programmes in the community. We estimated time trends in blood pressure (BP), anthropometric variables and smoking in the Vietnamese population and highlighted the differences between men and women or between rural and urban areas.

**Methods:**

A dataset of 23,563 adults aged 25–74 from 5 cross-sectional surveys undertaken within Vietnam from 2001 to 2009 by the Vietnam National Heart Institute was used to estimate mean BP, weight, waist circumference (WC), body mass index (BMI), the prevalence of hypertension, adiposity or smoking, which were standardised to the national age structure of 2009. Multilevel mixed linear models were used to estimate annual changes in the variables of interest, adjusted by age, sex, residential area, with random variations for age and surveyed provinces.

**Findings:**

Among the adult population, the age-standardised mean systolic and diastolic BP increased by 0.8 and 0.3 mmHg in women, 1.1 and 0.4 mmHg in men, while the mean BMI increased by 0.1 kgm^−2^ in women, 0.2 kgm^−2^ in men per year. Consequently, the prevalence of hypertension and adiposity increased by 0.9 and 0.3% in women, 1.1 and 0.9% in men with similar time trends in both rural and urban areas, while smoking prevalence only increased in women by 0.3% per year. A U-shaped association was found between age-adjusted BP and BMI in both sexes and in both areas.

**Conclusions:**

From 2001 to 2009, mean BP, weight and WC significantly increased in the Vietnamese population, leading to an increased prevalence of hypertension and adiposity, suggesting the need for the development of multi-sectoral cost-effective population-based interventions to improve CVD management and prevention. The U-shaped relationship between BP and BMI highlighted the hypertension burden in the underweight population, which is usually neglected in CVD interventions.

## Introduction

Three recent analyses of the trends in systolic blood pressure (BP), body mass index (BMI) and serum total cholesterol over the past generation raised concern about a global epidemic of risk factors for cardiovascular diseases (CVD) and called for actions to avoid catastrophic outcomes, especially in low and middle income countries (LMICs) where 80% of the total CVD burden occurs [1], [2], [3], [4]. However, the calculations for developing countries contained some uncertainties due to the lack of longitudinal, national-level, high quality, standardised or comparable surveys from LMICs. In Vietnam, a number of population-based surveys in different areas of the country showed an increasing prevalence of hypertension, obesity and smoking but none assessed the time trends of these major CVD risk factors [5], [6], [7]. Using data from published and unpublished studies, a comprehensive individual participant-level dataset from 2001 to 2009 was collated. Taking into account the variations in age patterns and geographical areas for each component survey, we estimated the time trends in BP, BMI, and smoking status in adult Vietnamese population over a 9-year period and highlighted the differences between men and women as well as the differences between urban and rural areas. These findings will be important for policy planners to coordinate the intervention strategies required to reduce the emerging CVD burden in Vietnam.

## Methods

### Study Population and Data Source

The integrated individual participant-level dataset used in this study came from several population-based cross-sectional surveys with similar designs and protocols from 2001 to 2009. All studies were designed and carried out by the Vietnam National Heart Institute (VNHI). In total, five datasets were used for this study and included the following surveys:

- The national epidemiological survey on hypertension and its risk factors in 8 provinces around Vietnam from 2001 to 2008 (NESH) [7]: multi-stage sampling strategies were applied to randomly select 110 participants from each commune, 3 communes from each district and 4 districts from each province. In total 9,823 participants (overall response rate of 93.0%) were recruited from 8 provinces (including 4 northern provinces: Hanoi, Thai Binh, Thai Nguyen, Nghe An, and 4 southern provinces: Khanh Hoa, Dac Lac, Dong Thap and Ho Chi Minh City).
- The survey on heart failure and its risk factors in 4 northern provinces of Vietnam from 2003 to 2004 (HF-S) (*unpublished data*): a similar sampling strategy to NESH was applied to select 4,840 participants (overall response rate of 91.7%) in 4 northern provinces of Vietnam: Hanoi, Thai Binh, Thai Nguyen and Nghe An.
- The survey on diabetes and its risk factors from 2008 to 2009 in 2 northern provinces of Vietnam (DM-S) [8]: a similar sampling strategy to NESH was applied to select 2,306 participants (overall response rate of 87.3%) in 2 northern provinces of Vietnam: Hanoi, Thai Binh.
- The survey on non communicable disease risk factors in FilaBavi in 2005 (NCDS) [5]: stratified random sampling was applied to select 250 people in each sex and 10-year group, which followed WHO’s STEPwise approach [9] and was based on the FilaBavi sampling frame [10]. Total 2,362 participants (overall response rate of 94.5%) were recruited from Ba Vi District of Ha Tay province.
- The screening surveys at all communes from the project on hypertension management in rural communes (HMPS) before 2010 when the National Target Project on Prevention and Control of Hypertension was started in Vietnam (*unpublished data*): in these surveys, simple random sampling strategy was applied to select participants from a list of all local inhabitants at the moment of the surveys. Total 5,855 participants (overall response rate of 97.6%) were recruited. These datasets included the pre-intervention cross-sectional surveys at Xuan Canh commune of Dong Anh District, Hanoi in 2004 and at Phu Cuong commune of Ba Vi District, Ha Tay province in 2006. It also included the cross-sectional surveys in 2007 and 2009 at Phu Phuong and Phu Cuong communes, which were control and intervention communes in a quasi-experimental study in Ba Vi District, Ha Tay Provinces.

To maximize comparability across the 5 surveys, we restricted analyses to examined, non-pregnant adults who were aged 25 to 74 years. This consisted of 9,403, 4,494, 2,098, 2,357 and 5,210 persons or 95.7%, 92.9%, 91.0%, 99.8% and 89.0% of studied samples in the 5 datasets, respectively.

### CVD Risk Factor Assessments

In all surveys, physical measurements such as BP, weight, height, waist circumference (WC) and hip circumference were taken using the same standardised protocol which has been described elsewhere [7]. BP was measured at least twice in a resting, sitting position using an automatic digital sphygmomanometer (OMRON Healthcare Inc.®, Bannockburn, Illinois), with an appropriately sized cuff. Hypertension was defined as an average systolic BP (SBP) ≥140 mmHg, and/or average diastolic BP (DBP) ≥90 mmHg, and/or self-reported current treatment with antihypertensive medications [11], [12]. Awareness of hypertension was defined as self-report of any prior diagnosis of hypertension by a health care professional. Treatment of hypertension was defined as use of a prescription medication for management of high BP at the time of the interview. Controlled hypertension was defined as pharmacological treatment of hypertension associated with an average SBP<140 mmHg and DBP<90 mmHg.

All other anthropometric measurements were performed at least twice with the participants wearing light clothing and no footwear. BMI was calculated as weight (kg) divided by height squared (m^2^). In this study, we used a BMI cut-off point of <18.5 kgm^−2^ for underweight, 18.5–22.9 kgm^−2^ for normal range, ≥23 kgm^−2^ for overweight, and ≥25 kgm^−2^ for generalized obesity. Abdominal obesity was defined as WC either ≥90 cm in men or ≥80 cm in women. Adiposity was defined as having either generalized obesity or abdominal obesity or both. These criteria have been specified for South-Asian populations by WHO Regional Office for Western Pacific [13], [14]. People who smoked tobacco products such as cigarettes, cigars or pipes over the previous month were classified as current smokers. Urban or rural residential area was identified on an administrative basis for each commune within each province.

### CVD Risk Factor Measurements in Studied Population

The prevalence of hypertension and obesity as well as the mean levels of individual BP and anthropometry measurements for each year between 2001 to 2009 were weighted and age-standardised for both sexes and residential areas, using age distribution data for related provinces from the Vietnam Population and Housing Census in 2009 [15].

Estimation of the annual incidence of hypertension was based on annual trends in consecutive prevalence of hypertension in the population. Using demographic data for the Vietnamese population structure and age distributions from the General Office for Population and Family Planning [16] and the age-standardised surveyed prevalence of hypertension from studied samples for each year during the period from 2001 to 2009, we estimated the number of people with hypertension among the general adult population aged 25–74. Assuming that hypertension is a life-long progressive disease until death; the prevalence of hypertension was very low at the age of 25 years; the general population changed consistently every year and hypertension-related case-fatality was constant, the difference between two consecutive values for current cases of hypertension in the population was used to extrapolate the number of new cases of hypertension every year. Then we estimated the annual average number of new cases of hypertension during the period 2001–2009 and the average population at risk from 2001 to 2009 to calculate the average annual incidence of hypertension among the adult population aged 25–74 years, separately for each sex and each residential area.

### Time Trends Analysis

The prevalence of hypertension, adiposity and smoking were aggregated and analysed by 3-year periods (2001 to 2003, 2004 to 2006, and 2007 to 2009) to yield clearer time trends across sex and residential areas. For the visual demonstrations of trends across time, we plotted trends using linear fit plots plus smooth fractional-polynomial fit plots based on the age-adjusted mean of relevant variables over surveyed years with corresponding estimated 95% confidence intervals (CI).

The unadjusted p-value for trend was calculated using univariable linear regression analysis (function *regress* in STATA) for each relevant variable, using year as a single continuous independent variable. For the multivariable analyses of the time trends based on all individual-level observations, we modelled trends using multilevel mixed-effect linear regression (function *xtmixed* in STATA), fitted via maximum likelihood, containing both fixed-effects and random-effects parts in order to compensate the potential heteroskedasticity among component surveys. Using the variables of studied year (treated as a continuous variable), participant’s age, sex and residential areas for the fixed-effects, this part allowed estimation of the changes in relevant variables by year after adjusting for age, sex and residential areas. Taking into account the fact that each survey was carried out in a specific geographical area (province or district) at a specific time of year in a selected sample representative of a specific population with possible different variations, the random-effects were random slope models adjusted for age and surveyed year and grouped by the studied provinces. For example, the mixed-effect model for SBP (model 1) was: SBP  = α+β1×survey year + β2×age + β3×sex + β4×residential area + Z× province× (b1×survey year + b2×age). The value of β1 in model 1 is the estimated annual mean change in SBP (or annual change in other relevant variables in other similar models) after adjusting for age, sex, and residential area with random effect part of Z grouped by each province at a random slope (b1 and b2 were the intercepts for age and surveyed year). A significant β coefficient (i.e. its 95% CI did not cover zero) with corresponding p-value (adjusted p for trend) suggested a significant trend over time.

To highlight the difference between women and men, we used similar models separated by sex or the original models with the addition of a sex-by-survey year interaction term as an independent variable, like the following model (model 2 for SBP for example): SBP  = α+β1×survey year + β2×age + β3×sex + β4×residential area + β5× (sex×survey year) + Z×province× (b1×survey year + b2×age). A negative coefficient for the interaction term β5 suggested a negative association between sex and SBP (or other relevant variable) over calendar years (i.e. a decrease in the slope). A positive coefficient suggested a positive association. A statistically significant p-value for the coefficient for the interaction (i.e. its 95% CI did not cover zero) suggested a difference in time trends between two sexes. Similar models including interaction analysis were applied to highlight the difference in time trend between urban and rural areas.

### Statistical Analysis

A p-value <0.05 was considered to represent statistical significance. Both descriptive and analytical statistical analyses were carried out using STATA 11 software (Stata Corporation®, Texas, USA).

### Ethical Statement

The protocols of each survey were all approved by both Scientific Ethical Committees in Biomedical Research at Vietnam National Heart Institute and the ones at the corresponding involved partners. All human subjects in any surveys were asked for their written consent before collection of data, and all had complete rights to withdraw from the study at any time without any threat, or disadvantage. Any participants with hypertension or other disorders were referred to appropriate facilities for further investigation and treatment.

## Results

Our analysis included 23,563 adults aged 25–74, or 93.6% of the collated dataset (after excluding 1,624 cases aged over 74 or who were pregnant). All surveys used province-based sampling, in which multi-stage cluster sample surveys (NESH, HF-S, DM-S) provided 68% of all data, stratified random sampling (NCDS) provided 10% while simple random sample surveys (HMPS) provided 22% of all data (Table 1). Each year from 2001 to 2009 contained 9–10% of the data except for 2003 (18%) and 2004 (14%). In the final collated dataset, 61.7% of participants lived in rural areas. The proportion of men was 36–41% in almost all surveys (except for NCDS, which applied a sex-stratified sampling strategy) due to the lower proportion of men in rural populations, resulting from working-age migration (especially men to urban areas in Vietnam (Table 1)).

**Table 1:** General characteristics of subpopulations aged 25–74 from multiple cross-sectional community surveys from 2001 to 2009 around Vietnam.

				Subpopulation aged 25–74, extracted for this study††				
No	Original Dataset	Total samplesize	Sampling strategy of each survey	Study period	N (%)†††	Men (%)*	Age (Mean±SD)*	Residence Rural (%)*
01	NESH - The national epidemiological surveyon hypertension and its risk factors	9,823	- Multi-stage: 110 people per commune, 3 communesper district, 4 districts per province. −8 provinces†:HN, TB, NA, TN, KH, DL, DT, HC.	2001–2008	9,403 (95.7%)	39.4	45.0±12.4	59.2
02	HF-S - The survey on heart failure andits risk factors	4,840	- Multi-stage: similar to NESH. −4 provinces†:HN, NA, TB, TN.	2003–2004	4,494 (92.9%)	41.3	45.7±12.8	59.0
03	DM-S - The survey on diabetes andits risk factors	2,306	- Multi-stage: similar to NESH. −2 provinces†:HN, TB.	2008–2009	2,098 (91.0%)	35.9	49.9±12.3	43.1
04	NCDS - The survey on non-communicabledisease risk factors	2,362	- Stratified random sampling: 250 individuals in eachsex and 10-year age group usin*g* FilaBavi samplingframe† −1 district: Bavi, HT province†	2005	2,357 (99.8%)	48.2	49.3±14.2	100
05	HMPS - The hypertension managementprogramme in rural communes	5,855	- Simple random selection. −3 communes: (controlor pre-intervention): XC from HN, PC and PP fromBavi District, HT†	2004–2009	5,210 (89.0%)	38.1	47.8±12.7	58.8

† DL: Dac Lac; DT: Dong Thap; FilaBavi: Demographical Surveillance System in Bavi District, Ha Tay Province; KH: Khanh Hoa; HC: HoChiMinh City; HN: Hanoi; HT: Ha Tay; NA: Nghe An; PC: Phu Cuong; PP: Phu Phuong; TB: Thai Binh; TN: Thai Nguyen; XC: Xuan Canh.

†† Exclude people over 75, pregnant women;

††† Proportion of original sample.

* Significantly different among original surveys with Chi-square test (p<0.001).

### Time Trends between Men and Women

The yearly variations in age-standardised mean BP (both SBP and DBP), weight, WC, BMI, and the age-standardised prevalence of hypertension (as well as prevalence of awareness, treatment and control of hypertension), adiposity (both generalized and abdominal obesity) and current smoking are summarized in Table 2 and Table 3, stratified by sex, and residential areas. The diversification by year for each variable of interest shown in Table 2 and 3 reflected the real differences of general characteristics of potentially different studied population at certain specific time or area.

**Table 2:** Trends in mean age-standardised systolic and diastolic blood pressure, weight, waist circumference, body mass index and waist-hip ratio in Vietnam population aged 25–74 between 2001 and 2009, stratified by sex and residential areas.

			Systolic Blood Pressure (mmHg)		Diastolic Blood Pressure (mmHg)		Weight (kg)		Waist Circumference (cm)		Body Mass Index (kgm^−2^)		Waist-Hip Ratio	
Year	Sample size	Survey †	Women	Men	Women	Women	Women	Men	Women	Men	Women	Men	Women	Men
**Rural area (mean±SD)**														
2001	1,093	(1)	117.1±14.6	119.7±16.0	73.1±10.0	74.3±10.7	45.3±6.0	51.5±6.5	67.0±5.9	70.0±6.0	19.5±2.2	19.7±2.0	0.82±0.05	0.84±0.05
2002	1,996	(1)	114.9±15.2	118.5±15.6	72.0±9.8	73.9±10.4	44.4±5.7	51.0±6.2	65.5±6.4	69.1±6.4	19.4±2.1	19.6±2.0	0.79±0.05	0.81±0.05
2003	2,512	(1)+(2)	117.8±16.8	121.3±17.4	75.5±10.5	78.1±11.3	45.2±6.6	51.6±6.7	66.2±8.8	68.8±7.1	19.7±2.4	19.9±2.2	0.81±0.08	0.83±0.06
2004	889	(2)	114.4±15.8	117.9±16.7	72.9±9.1	74.6±10.0	46.0±6.2	52.9±6.4	66.4±6.3	69.7±5.5	19.8±2.2	20.2±2.0	0.81±0.05	0.84±0.05
2005	2,357	(4)	117.2±17.1	125.9±17.7	71.2±10.6	76.0±11.6	45.0±6.2	51.2±6.8	68.5±6.7	71.4±6.7	19.4±2.3	19.5±2.2	0.81±0.05	0.83±0.05
2006	1,923	(1)+(5)	123.8±18.2	132.1±16.5	76.7±11.2	80.3±10.6	47.1±6.5	54.5±7.4	66.9±6.3	70.7±6.5	20.5±2.5	20.8±2.3	0.83±0.05	0.87±0.05
2007	1,831	(1)+(5)	121.3±18.4	129.5±18.3	76.7±10.5	80.4±11.5	50.6±8.1	56.3±7.7	72.8±9.2	73.9±8.2	21.2±3.1	20.8±2.6	0.85±0.07	0.86±0.06
2008	–	–	–	–	–	–	–	–	–	–	–	–		
2009	1,946	(3)+(5)	120.9±18.2	129.6±18.7	74.6±10.8	79.1±11.6	46.2±5.9	53.7±7.2	70.3±6.5	73.7±7.2	19.9±2.3	20.5±2.3	0.83±0.05	0.85±0.06
*p for trend* *			<0.001	<0.001	<0.001	<0.001	<0.001	<0.001	<0.001	<0.001	<0.001	<0.001	<0.001	<0.001
**Urban area (mean±SD)**														
2001	1,293	(1)	116.4±19.5	121.9±16.8	74.3±11.8	78.5±11.0	51.4±7.7	58.3±8.7	71.1±8.2	74.7±8.3	21.9±3.0	21.7±2.8	0.82±0.07	0.86±0.06
2002	598	(1)	117.3±19.0	121.3±16.2	73.2±11.4	75.7±11.1	48.3±7.5	54.3±7.6	70.4±7.6	74.1±7.5	21.0±2.8	20.9±2.4	0.80±0.05	0.83±0.06
2003	1,830	(1)+(2)	116.1±18.1	123.3±18.6	73.4±10.5	78.3±11.4	51.2±7.5	57.2±8.7	72.7±7.7	74.6±8.1	21.9±2.9	21.6±2.8	0.85±0.07	0.88±0.06
2004	2,427	(2)+(5)	118.0±16.6	125.3±17.4	75.9±10.1	80.9±11.3	47.9±6.5	55.1±8.0	70.0±6.9	73.7±7.3	20.8±2.5	21.0±2.5	0.83±0.06	0.87±0.06
2005	–	–	–	–	–	–	–	–	–	–	–	–	–	–
2006	237	(1)	122.9±21.3	133.3±23.5	74.0±11.1	79.1±14.6	48.8±6.8	54.7±6.7	68.5±7.0	71.2±6.3	21.4±2.8	21.0±2.4	0.84±0.07	0.87±0.05
2007	284	(1)	127.1±22.4	128.0±16.2	79.5±13.1	80.8±11.7	51.1±9.4	54.5±7.9	77.2±9.1	74.1±8.2	21.3±3.0	20.2±2.4	0.90±0.06	0.87±0.06
2008	2,058	(1)+(3)	123.1±20.1	130.1±19.5	77.1±11.8	82.0±12.0	53.6±8.6	59.8±10.6	75.1±8.4	77.3±9.6	22.8±3.4	22.4±3.4	0.86±0.06	0.88±0.07
2009	288	(3)	119.2±18.2	129.6±19.9	74.4±10.5	78.7±12.4	49.6±6.3	57.4±9.3	73.6±7.0	76.6±8.3	21.4±2.4	21.6±3.1	0.84±0.06	0.87±0.05
*p for trend* *			<0.001	<0.001	<0.001	<0.001	<0.001	<0.001	<0.001	<0.001	<0.001	<0.001	<0.001	<0.001

† (1) NESH: the national epidemiological survey on hypertension and its risk factors; (2) HF-S: the survey on heart failure and its risk factors; (3) DM-S: the survey on diabetes and its risk factors; (4) NCDS: the survey on non-communicable disease risk factors; (5) HMPS: the hypertension management programme in rural communes (naïve communes).

* p for trend was estimated from univariable linear regression between the variable of interest with time (year).

**Table 3:** Trends in age-standardised prevalence of hypertension, adiposity, and current smoking in Vietnam population aged 25–74 between 2001 and 2009, stratified by sex, and residential areas.

			Hypertension (BP≥140/90 mmHg or currently treated)										Adiposity			
			Evidence of hypertension		Awareness of hypertension††		Treated hypertension††		Controlled hypertension††		Current Smoking		Obesity BMI ≥25 kgm^−2^		Abdominal fatness†††	
Year	Sample size (n)	Survey†	Women	Men	Women	Men	Women	Men	Women	Men	Women	Men	Women	Men	Women	Men
**Rural area (%)**																
2001	1,093	(1)	9.4	12.3	11.4	17.6	4.1	3.5	1.3	0.6	0.6	63.7	1.3	1.6	3.8	0.8
2002	1,996	(1)	6.9	11.4	23.1	30.2	0.6	0.9	0.0	0.0	0.5	60.0	2.0	1.7	2.8	0.5
2003	2,512	(1)+(2)	13.0	21.1	11.1	9.1	4.0	3.9	1.2	1.1	2.1	65.4	3.2	2.8	9.2	1.0
2004	889	(2)	8.8	11.9	4.3	3.5	2.6	0.0	2.6	0.0	1.3	53.5	2.2	1.7	4.7	0.2
2005	2,357	(4)	11.2	19.6	24.2	19.2	9.2	5.9	4.6	1.0	0.8	61.5	1.6	1.9	6.9	1.3
2006	1,923	(1)+(5)	17.7	31.7	32.1	18.6	28.5	15.3	9.0	7.3	16.6	70.6	5.7	4.6	3.9	1.2
2007	1,831	(1)+(5)	23.3	25.8	70.2	51.7	65.1	46.8	41.1	18.7	1.5	63.2	11.0	8.2	22.4	5.3
2008	–	–	–	–	–	–	–	–	–		–	–	–	–	–	–
2009	1,946	(3)+(5)	15.3	24.4	30.6	26.8	19.8	19.7	2.9	3.3	2.0	61.6	2.4	3.0	7.6	2.5
*p for trend* *			<0.001	<0.001	<0.001	<0.001	<0.001	<0.001	<0.001	<0.001	0.001	0.130	0.003	0.001	<0.001	<0.001
**Urban area (%)**																
2001	1,293	(1)	16.0	21.2	47.6	35.9	8.1	3.7	0.0	0.6	0.8	64.4	15.6	12.4	14.6	3.7
2002	598	(1)	16.0	15.4	46.8	34.4	4.1	1.2	4.1	0.0	0.4	61.4	9.5	8.0	12.0	2.4
2003	1,830	(1)+(2)	14.8	23.8	32.1	19.4	14.4	2.6	6.2	0.1	1.8	64.5	15.1	13.8	15.4	3.5
2004	2,427	(2)+(5)	16.5	31.7	29.0	19.3	25.2	16.5	7.3	4.3	0.1	59.1	6.0	8.1	9.5	2.8
2005	–	–	–	–	–	–	–	–	–	–	–	–	–	–	–	–
2006	237	(1)	20.6	25.7	41.6	33.2	35.4	33.2	12.5	6.8	18.6	65.9	9.2	6.9	6.9	0.1
2007	284	(1)	31.6	40.4	59.7	63.1	55.1	60.6	16.5	31.1	5.2	69.3	9.0	4.7	35.5	5.3
2008	2,058	(1)+(3)	29.2	35.3	59.2	34.8	56.9	30.0	23.8	9.9	2.0	54.7	22.3	23.9	25.9	8.6
2009	288	(3)	16.1	32.7	53.8	42.6	39.6	37.2	14.2	17.4	3.8	56.6	8.6	18.2	16.5	5.3
*p for trend* *			<0.001	<0.001	<0.001	<0.001	<0.001	<0.001	<0.001	<0.001	<0.001	0.008	<0.001	<0.001	<0.001	<0.001

† (1) NESH: the national epidemiological survey on hypertension and its risk factors; (2) HF-S: the survey on heart failure and its risk factors; (3) DM-S: the survey on diabetes and its risk factors; (4) NCDS: the survey on non-communicable disease risk factors; (5) HMPS: the hypertension management programme in rural communes.

†† Prevalence in hypertensive population.

††† Waist circumference ≥90 cm in men or ≥80 cm in women.

* p for trend was estimated from univariable linear regression between the variable of interest with time (year).

**Figure 1:**
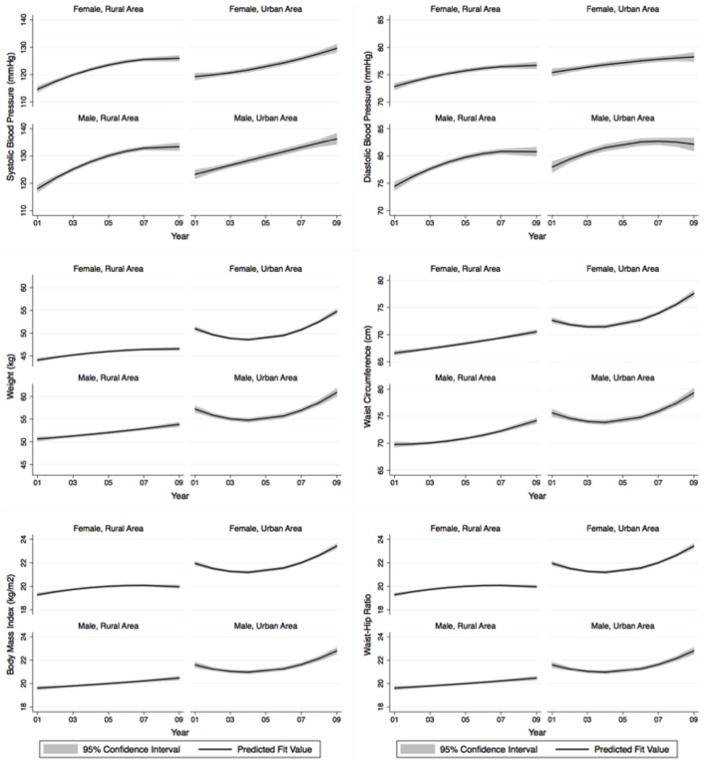
Trends in age-standardised mean of blood pressure (systolic and diastolic), weight, waist circumference, body mass index and waist-hip ratio between 2001 and 2008 for woman and men in rural and urban areas. The solid line represents fractional-polynomial prediction plots and the shaded area the 95% uncertainty interval.

**Figure 2:**
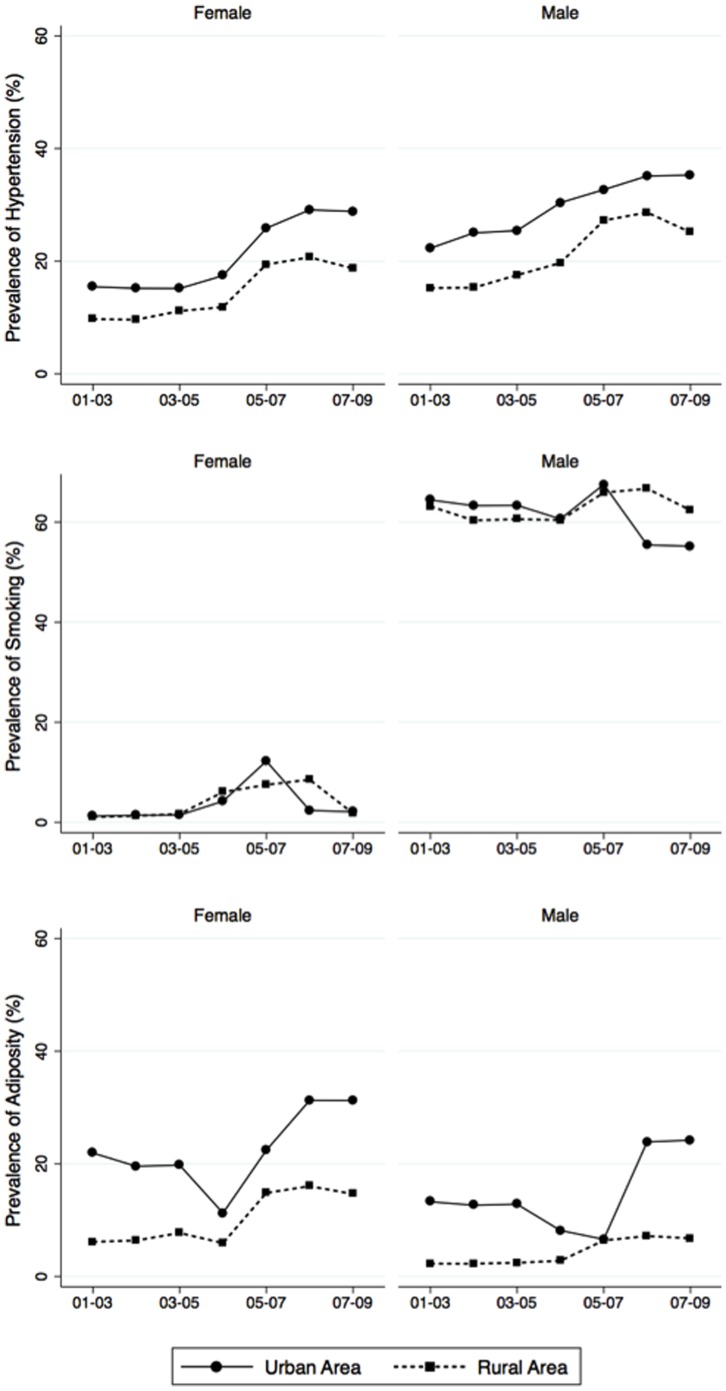
Changes over time in age-adjusted prevalence of hypertension, adiposity and smoking from 2001 to 2009 by urban and rural areas, demonstrated by 3-year moving averages.

Each year on average, age-adjusted mean systolic and diastolic BP increased 0.9 mmHg and 0.4 mmHg respectively in general population, at a considerably higher rate in men than in women (p<0.001). Consequently, during the period of 2001–2009, the prevalence of hypertension significantly increased by 0.9% per year in general adult population (p<0.001) with similar time trends between both sexes (p = 0.498). The mean BMI increased 0.1 kgm^−2^ annually in the adult population (p<0.001) with non-significant differences in time trends between both sexes (p = 0.589). However, the prevalence of abdominal obesity increased more rapidly than the prevalence of generalized obesity in women while this trend was reversed in men (Table 4). In general, except for the prevalence of current smoking in men (non-significant changes over time, p = 0.224), all other variables (either mean or prevalence) increased in a linear fashion from 2001 to 2009 in both sexes (Table 4, Figure 1 and Figure 2).

**Table 4:** Adjusted annual changes of CVD risk factors, stratified by sex.

	Overall annual changes		Annual changes in female		Annual changes in male		Sex-Year interactions	
Major CVD risk factors in population aged 25–74	Adjustedcoefficient95%CI	p fortrends	Adjustedcoefficient95%CI	p fortrends	Adjustedcoefficient95%CI	p fortrends	Adjustedcoefficient95%CI	p for different timetrends betweenfemale and male
Mean systolic blood pressure (mmHg)	0.890.76–1.02	<0.001	0.790.64–0.95	<0.001	1.090.88–1.30	<0.001	0.380.18–0.57	<0.001
Mean diastolic blood pressure (mmHg)	0.360.29–0.44	<0.001	0.330.23–0.42	<0.001	0.440.32–0.57	<0.001	0.220.10–0.34	<0.001
Prevalence of hypertension (%)	0.900.62–1.17	<0.001	0.850.52–1.18	<0.001	1.120.65–1.59	<0.001	0.15(−0.28)−0.57	0.498
Prevalence of aware hypertension (%)†	0.51(−0.13)−1.14	0.116	0.46(−0.40)−1.31	0.292	0.77(−0.15)−1.68	0.100	(−0.58)(−1.54)−0.37	0.234
Prevalence of treated hypertension (%)†	3.292.75–3.82	<0.001	3.402.66–4.14	<0.001	3.302.55–4.06	<0.001	(−1.01)(−1.82)−(−0.20)	0.015
Prevalence of controlled hypertension (%)†	0.650.30–1.00	<0.001	0.610.09–1.13	0.020	0.820.40–1.24	<0.001	(−0.93)(−1.46)−(−0.41)	<0.001
Mean weight (kg)	0.350.30–0.41	<0.001	0.270.21–0.33	<0.001	0.520.43–0.60	<0.001	0.06(−0.02)−0.13	0.167
Mean waist circumference (cm)	0.460.41–0.52	<0.001	0.450.39–0.51	<0.001	0.510.43–0.60	<0.001	(−0.01)(−0.09)−0.07	0.850
Mean body mass index (kgm^−2^)	0.100.08–0.12	<0.001	0.070.05–0.10	<0.001	0.160.13–0.19	<0.001	0.01(−0.20)−0.04	0.589
Mean waist−hip ratio (cm/cm)	0.00430.0039–0.0047	<0.001	0.00420.0037–0.0047	<0.001	0.00470.0040–0.0053	<0.001	(−0.0003)(−0.0009)−0.0003	0.308
Prevalence of adiposity (%)	0.490.27–0.71	<0.001	0.340.04–0.63	0.027	0.910.62–1.19	<0.001	0.16(−0.17)−0.49	0.345
Prevalence of generalized obesity (%)	0.340.17–0.52	<0.001	0.14(−0.09)−0.37	0.227	0.770.50–1.05	<0.001	0.24(−0.03)−0.51	0.083
Prevalence of abdominal fatness (%)	0.350.16–0.54	<0.001	0.470.19–0.75	0.001	0.310.13–0.49	0.001	(−0.28)(−0.56)−0.01	0.063
Prevalence of current smoking (%)	0.07(−0.16)−0.30	0.555	0.330.20–0.45	<0.001	−0.33(−0.87)−0.20	0.224	−0.92(−1.27)− (−0.57)	<0.001

* Estimated by multilevel mixed model linear regression, adjusted by age, surveyed year, residential area and grouped by provinces with random slop for year and age in each provinces.

† Among hypertensive population.

Using two consecutive extrapolations of the prevalence of hypertension in Vietnamese adults, the estimated annual incidence of hypertension among the population aged 25–74 was 65 per 10,000 in women and 165 per 10,000 in men, or the projected burden of hypertension in adult population of Vietnam was 140,000 new hypertensive women and 330,000 new hypertensive men every year (Table 5).

**Table 5:** Estimation of yearly incidence of hypertension based on the trends in age-standardised prevalence of hypertension, in Vietnam population aged 25–74 between 2001 and 2009, stratified by sex.

	Female population (n±95%SE)				Male population (n±95%SE)			
Year	Prevalence of hypertension (%)^1^	Population at risk (millions)^2^	People with high BP (millions)^3^	Estimated new cases (millions)^4^	Prevalence of hypertension (%)^1^	Population at risk (millions)^2^	People with high BP (millions)^3^	Estimated new cases (millions)^4^
2001	12.99±0.86	19.0	2.47±0.16		16.82±1.32	17.5	2.94±0.23	
2002	7.30±0.73	19.6	1.43±0.14	−1.04	11.60±1.08	17.9	2.08±0.19	−0.86
2003	13.94±0.80	19.7	2.75±0.16	1.32	22.37±1.17	18.3	4.09±0.21	2.01
2004	10.81±0.92	20.7	2.24±0.19	−0.51	16.71±1.34	18.8	3.14±0.25	−0.95
2005	11.16±0.88	20.4	2.28±0.18	0.04	19.65±1.26	19.5	3.83±0.25	0.69
2006	18.14±1.29	20.1	3.65±0.26	1.37	30.97±2.01	20.2	6.26±0.41	2.43
2007	24.35±1.53	22.6	5.50±0.35	1.85	27.37±1.99	20.8	5.69±0.41	−0.57
2008	29.24±1.32	23.5	6.87±0.31	1.37	35.26±2.20	21.7	7.65±0.48	1.96
2009	15.42±1.20	23.2	3.58±0.28	−3.29	25.75±1.98	21.6	5.56±0.43	−2.09
Mean		21.23		0.139		19.85		0.328
	Estimated yearly incidence of hypertension = 65±6/10,000^5^				Estimated yearly incidence of hypertension = 165±10/10,000^5^			

1 Age-standardised prevalence of hypertension from studied sample;

2 Number of people aged 25–74 from general population of Vietnam;

3 Estimation from (1) and (2);

4 New cases of hypertension estimated from two consecutive prevalence of hypertension;

5 Yearly incidence of hypertension estimated from average number of new cases and average population at risk every year.

### Time Trends between Urban and Rural Area

Mean systolic and diastolic BP in adult population both increased significantly in both rural and urban areas (p<0.001) with significant time trends between areas. High blood pressure was detected and controlled significantly more with similar time trends in both urban and rural areas (p<0.001) although the prevalence of controlled hypertension in rural areas (p = 0.253) and the prevalence of aware hypertension in urban areas (p = 0.568) did not change considerably over time (year). Other anthropometric indexes such as mean weight, WC, BMI or WHR significantly increased over time in both areas although all the rates (time trend slope) in rural areas were higher than in urban areas (all p<0.05). Consequently, the prevalence of adiposity rose significantly overtime in both areas although the prevalence of generalized obesity in rural areas (p = 0.063) and the prevalence of abdominal fatness in urban areas (p = 0.153) did not change in a clear linear fashion with time (Table 6).

Aggregated age-standardised prevalence in each triennium shows more clearly the sex-specific time trends of hypertension, adiposity and smoking in urban and rural area (Figure 2). Although there was a breakdown in trend of adiposity prevalence likely resulting from BMI variations in the surveyed provinces, overall the prevalences of hypertension and adiposity were higher in urban than rural areas and both prevalences trended upwards over time (Figure 2). While the time trends in smoking prevalence were similar between urban and rural areas (p = 0.546), smoking increased significantly over time among women (starting from a very low baseline) but did not reduce significantly among men (Figure 2 and Table 6).

**Table 6:** Adjusted annual changes of CVD risk factors, stratified by residential areas.

	Overall annual changes		Annual changes in rural		Annual changes in urban		Residential area-Year interactions	
Major CVD risk factors in populationaged 25–74	Adjustedcoefficient95%CI	p fortrends*	Adjustedcoefficient95%CI	p fortrends*	Adjustedcoefficient95%CI	p fortrends*	AdjustedCoefficient95%CI	p for different time trends between rural and urban*
Mean systolic blood pressure (mmHg)	0.890.76–1.02	<0.001	0.840.67–1.01	<0.001	0.960.77–1.15	<0.001	0.230.003–0.46	0.047
Mean diastolic blood pressure (mmHg)	0.360.29–0.44	<0.001	0.460.36–0.56	<0.001	0.250.14–0.36	<0.001	(−0.20)(−0.33)−(−0.06)	0.005
Prevalence of hypertension (%)	0.900.62–1.17	<0.001	0.760.42–1.10	<0.001	1.090.66–1.51	<0.001	0.41(−0.09)−0.91	0.105
Prevalence of aware hypertension (%)†	0.51(−0.13)−1.14	0.116	1.050.15–1.96	0.023	0.25(−0.61)−1.12	0.568	(−1.02)(−2.14)−0.09	0.073
Prevalence of treated hypertension (%)†	3.292.75–3.82	<0.001	2.892.13–3.65	<0.001	3.722.97–4.47	<0.001	0.80(−0.14)−1.75	0.096
Prevalence of controlled hypertension (%)†	0.650.30–1.00	<0.001	0.27(−0.19)−0.73	0.253	1.180.67–1.68	<0.001	0.13(−0.48)−0.74	0.673
Mean weight (kg)	0.350.30–0.41	<0.001	0.440.38–0.50	<0.001	0.260.17–0.34	<0.001	(−0.15)(−0.24)−(−0.06)	0.001
Mean waist circumference (cm)	0.460.41–0.52	<0.001	0.590.53–0.66	<0.001	0.310.22–0.39	<0.001	(−0.23)(−0.32)−(−0.14)	<0.001
Mean body mass index (kgm^−2^)	0.100.08–0.12	<0.001	0.130.10–0.15	<0.001	0.080.05–0.11	<0.001	(−0.04)(−0.07)−(−0.01)	0.016
Mean waist-hip ratio (cm/cm)	0.00430.0039–0.0047	<0.001	0.00560.0050–0.0061	<0.001	0.00280.0022–0.0035	<0.001	(−0.0012)(−0.0025)−(−0.0011)	<0.001
Prevalence of adiposity (%)	0.490.27–0.71	<0.001	0.410.19–0.64	<0.001	0.600.20–0.99	0.003	0.23(−0.17)−0.62	0.257
Prevalence of generalized obesity (%)	0.340.17–0.52	<0.001	0.16(−0.01)−0.33	0.063	0.590.24–0.94	0.001	0.340.02–0.66	0.038
Prevalence of abdominal fatness (%)	0.350.16–0.54	<0.001	0.400.20–0.60	0.001	0.25(−0.09)−0.60	0.153	0.06(−0.29)−0.39	0.772
Prevalence of current smoking (%)	0.07(−0.16)−0.30	0.555	0.06(−0.25)−0.37	0.678	0.13(−0.20)−0.46	0.455	0.13(−0.28)−0.54	0.546

* Estimated by multilevel mixed model linear regression, adjusted by age, surveyed year, sex and grouped by provinces with random slop for year and age in each provinces.

† Among hypertensive population.

**Figure 3:**
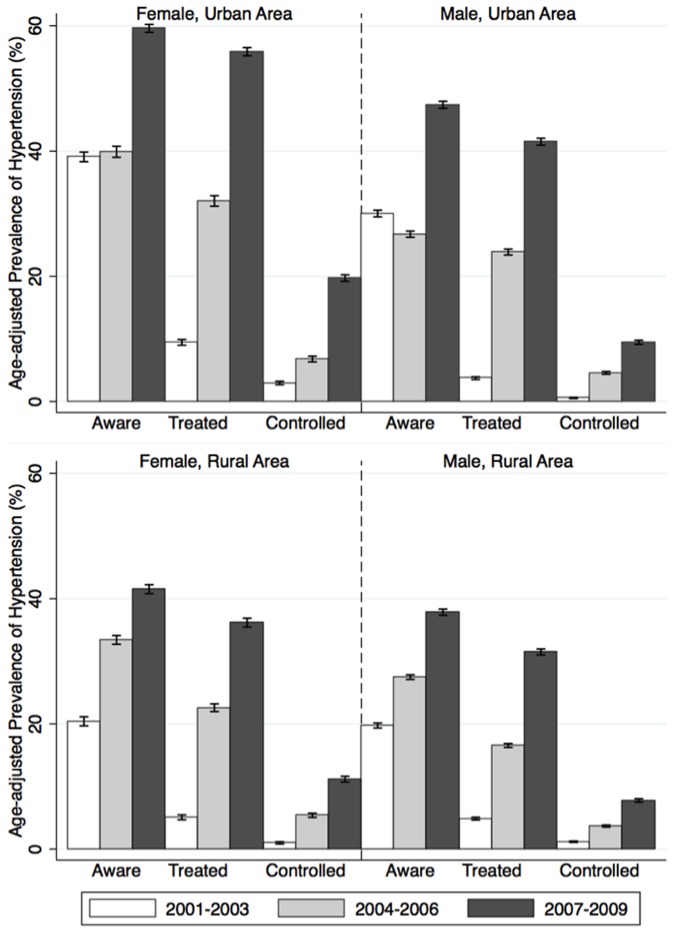
Change over time in age-standardised prevalence of awareness, treatment and control of hypertension by urban and rural areas.

The age-standardised prevalence of hypertension increased 0.9–1.0% per year, equally in both sexes and in both residential areas (Table 4 and 6), both of which arose mostly due to a shifting to the right of the BP distribution in the general population rather than small increases among hypertensives on treatment. Among hypertensives, the prevalence of awareness, treatment and control of hypertension were higher in women than in men and higher in urban than rural areas and all tended to improve over time (Figure 3). Even though the prevalences of treatment and control of hypertension increased significantly, there was obviously a big gap between the prevalence of hypertension (representing the demand) and the prevalence of treatment or control (representing the capacity) (Table 3 & Figure 3).

### U-shaped Relationship between BMI and Hypertension

**Figure 4:**
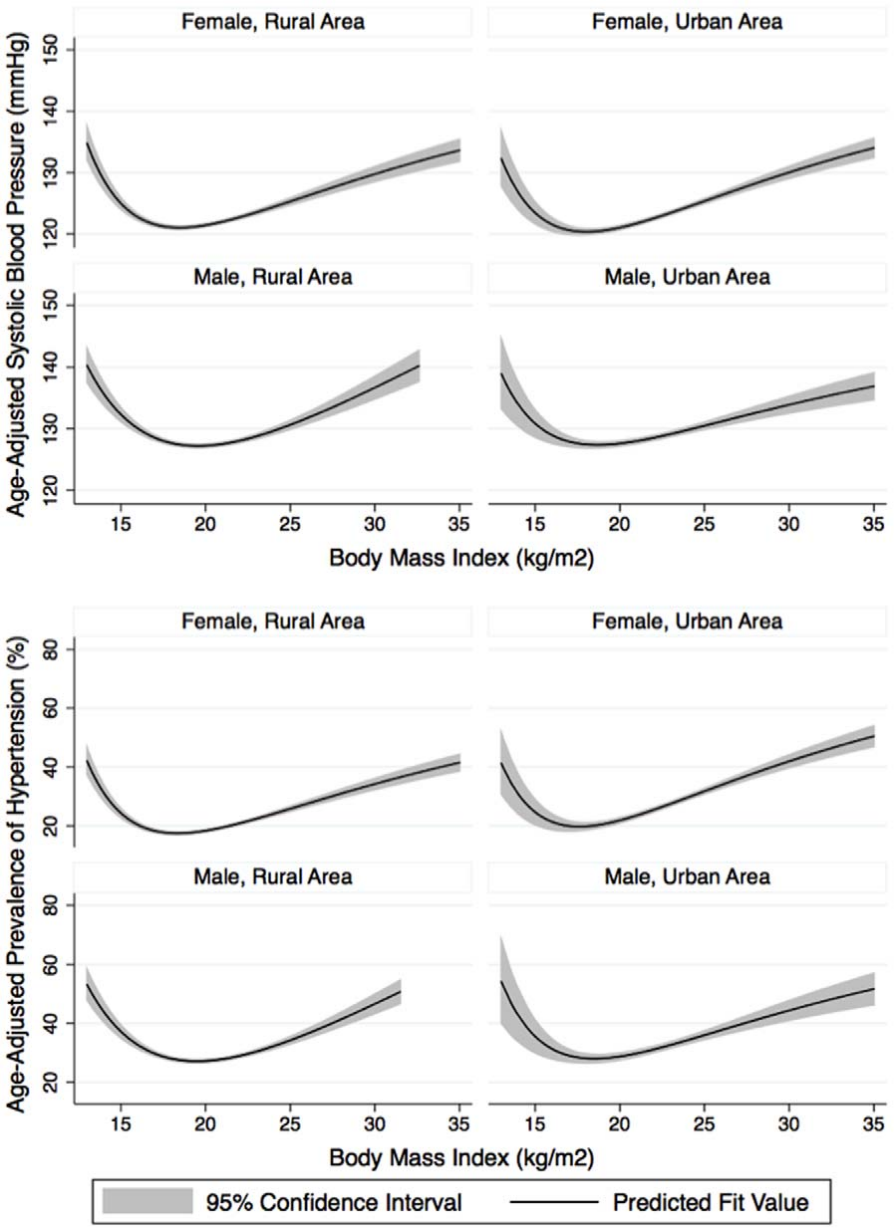
U-shaped relationship between age-adjusted systolic blood pressure or hypertension and body mass index, stratified by sex and residential areas. The solid line represents fractional-polynomial prediction plots and the shaded area the 95% uncertainty interval.

Analysing the relationship between BP and BMI, our data also showed that an increase in BMI was generally associated with a significant increase either in mean BP (both SBP and DBP) or prevalence of hypertension in an observable, linear fashion as BMI levels increased in both sexes. However, in the underweight population group, the age-standardised prevalence of hypertension significantly increased inversely with BMI in both sexes, making a U-shaped relationship between BMI and hypertension (Figure 4).

## Discussion

### Time Trends in Major CVD Risk Factors in Vietnam

Our results, based on data from multiple cross-sectional surveys across the country, demonstrated that the time trends in hypertension and adiposity, both major CVD risk factors, steadily increased from 2001 to 2009, confirming the global trends of SBP and BMI in the Vietnamese community and reflecting the contribution to increasing CVD mortality recorded in Vietnam and the region. [2], [3], [4], [17] However, using the available individual participant-level data in each year and taking into account large variations between the surveyed provinces, the estimated increases of mean SBP in our study were 0.8 mmHg per year in women and 1.1 mmHg per year in men, which far exceeded the global estimations of SBP increases in Southeast Asian region that ranged from 1.0–2.7 mmHg per decade in women and 0.8–1.6 mmHg per decade in men [3]. With similar trends of lower magnitude, our results estimated the increases of average BMI were 0.7–1.6 kgm^−2^ per decade in women and men in Vietnam while the mean global BMI only increased by 0.4–0.5 kgm^−^^2^ per decade in women and men together with the fact that BMI increased most slowly in the Southeast Asia region [2]. These trends in CVD risk factors at the population level, without rapid and widespread appropriate reactions, would lead to trends in CVD events in the same direction, threatening a projected CVD epidemic after a time lag of about 3–4 years [18].

The time trends also highlighted the bigger gaps between the potential burden of CVD risk factors and the real awareness of these and the capacity to solve them in the community (such as the hypertension issue demonstrated in Table 3, Table 6 & Figure 3), especially in rural areas, where there were unfavourable factors such as poorer access to medical services, inadequate supply of essential drugs, less health education and lower income [19]. So far, LMICs including Vietnam have a dearth of capacity to effectively deal with the existing and projected burden of cardiovascular diseases when health care systems are facing the challenges of reform to cope with the double burden of both relevant communicable and emerging non-communicable diseases [20]. On the other hand, in developed countries, CVD mortality/morbidity have reduced rapidly and substantially in recent decades, following both improvements in cardiac treatment and decreases in some but not all major CVD risk factors (such as blood pressure, cholesterol, and smoking) [2], [3], [4], [21]. These facts suggest that among the efforts to stem and reverse the tide of major CVD risk factors [17], endeavours to control blood pressure, smoking and cholesterol should be the prime concern. In low-resource settings like Vietnam, more resources should be allocated for cost-effective population-level interventions targeted at some similar major CVD risk factors (such as tobacco cessation or salt reduction to control BP) with multi-dimensional approaches (such as health education to promote healthy lifestyles in the population or policy changes to create favourable social environments) [2], [21], [22]. In addition, the existing healthcare system should be improved to promote efficient access to local healthcare services and be integrated with comprehensive surveillance systems so that any later interventions in local areas can be actively implemented, closely monitored and thoughtfully evaluated. Consequently any cost-benefit interventions can be tailored to tackle both communicable and non-communicable diseases at either community or individual level.

### Limitations of this Study

Using multi cross-sectional surveys with different sampling strategies and diverse geographical areas (provinces) and times during each year, our findings must be considered within the context of these limitations. Similar to other population-based cross-sectional surveys on hypertension and CVD risk factor, hypertension status may have been overestimated when it was assessed only during a single visit in the survey [23] and the overweight status may have been underestimated depending on harvest cycles, especially in rural areas. However, this should have minimal effects on within-sample comparisons and it is an inherent problem of large epidemiological investigations. Using a standard protocol with strong quality control procedures for any physical measurement and questionnaire by nearly identical core teams from VNHI for all surveys [7], the potential protocol-related heterogeneity and data-driven bias should have been minimized; consequently, the data variations could be explained mostly by the effects of time (year) and geographical area (province). In order to manage the heteroskedasticity across all component surveys, multilevel mixed linear modelling with random slope at province level for age and surveyed year was used to adjust the random variations within geographic areas and specific time of each year as well as the random variations of relevant variables with age. Moreover, age-standardised prevalences or means were used to compensate for the potential bias due to differences of age structure in each dataset. The availability of real individual observation-level data almost in every year of analysis rather than using assumed equations allowed us to capture more variation to reflect population health and to estimate annual changes more accurately. However, due to specific aims or protocols of the component surveys and their budget constraints, some yearly data were missing in particular areas (such as data in urban areas for 2005 or in rural areas for 2008), which could possibly increase the uncertainty of our estimations. For the same reason, our study also could not explore other CVD risk factors, their interactions or adjust for more potential confounders which were not fully collected in all component studies.

### U-shaped Interaction between Obesity and Hypertension

Although obesity and hypertension are considered as major independent CVD risk factors [24], [25], the interactions between these two factors and their impacts on CVD outcomes are complex [26], [27]. Our results revealed a U-shaped relationship between SBP or prevalence of hypertension and BMI in both sexes in the general population (in both urban and rural areas). In hypertensives, the CVD outcomes became excessive when patients were too thin or too fat, suggesting a similar curvilinear shape in relationship between CVD mortality and BMI [28], [29]. However, the hypothetical paradoxical link between BMI and CVD outcomes in the lean population group reflected in most cross-sectional surveys [30], including ours, does not compete against the demonstrated effectiveness of weight reductions in overweight populations or the impacts of significant weight gain on prevalence or incidence of CVD risk factors (such as hypertension, diabetes), and CVD events (including cardiac death) [26], [31], [32], [33], [34], [35], [36]. The data still support the need for weight reduction in the prevention and treatment of CVDs but further well-conducted studies with long-term follow-up are necessary to examine the effects of weight loss or weight gain on hypertension outcomes due to the complexity of the weight/hypertension relationship. Our data also highlighted the important and potential CVD burden of underweight status, which is often neglected in non-communicable disease areas but which is still widely prevalent in LMICs at early stages of epidemiologic transition where there is huge cumulative exposure to poverty and diseases, and nutritional deprivation [30].

### Conclusions

Over 9 years from 2001 to 2009, blood pressure, weight and waist circumference increased steadily in the adult population of Vietnam in both sexes and in both urban and rural areas, resulting in a growing epidemic of hypertension and obesity while the awareness, treatment and control of hypertension lagged far behind the prevalence. Concurrently, the prevalence of smoking increased in women while hardly decreasing in men. In addition to the interaction between obesity and hypertension, this study also highlights the potential burden of hypertension in the neglected underweight subpopulation. Altogether, the sheer volume of prospective cardiovascular diseases accruing from modifiable CVD risk factors indicates that a higher priority for both swift and effective population-based cardiovascular health interventions is needed.
